# Supplementary material for: Perceived stigma and depression among the HIV-positive adult people in Ethiopia: A systematic review and meta-analysis
Source: PLoS One. 2024 Jun 3;19(6):e0302875. doi: 10.1371/journal.pone.0302875 (PMC11146698; doi:10.1371/journal.pone.0302875)
Supplement: Attachment — Submitted filename: Response to reviewers .docx [file pone.0302875.s004.docx]

| Comments raised by | Comments raised | Authors response |
| --- | --- | --- |
| Editors | No comments. | Thank for you positive and building comments and suggestion. |
| Reviewer #1: | - The abstract section needs intensive revision and, - the recommendations should be based on the findings. - The abstract seems grammatically problem and, - I think the software STATA 16 version is not done currently how to use this software and, - Please rewrite the abstract section based on Plose journal format | ***Thank you for your building comments.***  **Revised!!!!**   - **Revised!!!!** - Yes of course BUT the meta-analysis was done before the STATA version 16 was stopped.   **Revised!!!!** |
|  | Just focus on Pooled prevalence not sample size only,  Neither the figure nor the table were watched. | **Thank you** for your insight comments.   - Regarding the figures since the figure is in the form of tiff file you can look by maximizing the tiff image and, - also I have tried to adjust in large font BUT converter brought the same TIFF image. |
|  | The conclusion should clearly show the major findings and the authors are expected to recommend based on modifieable variables. | **Thank you for your insight comments.**  **Revised!!!!** |
| Reviewer #2: | I would advise the authors to specify a specific start year for their search instead of saying "from inception." | **Thank You** for your encouraging constructing comments!!!!  **Revised!!!!** |
|  | Which institutions are the authors referring to? | **Thank You for your comments.** it is revised!! “originally Institution used to refer where the studies conducted” BUT now we have removed it poorly explains the conditions. |
|  | In Table 1, try to be consistent by either reporting the numbers to the nearest tens or hundreds. | **Thank You.**  for your encouraging constructing comments!!!!  **Revised!!!!** |
