# Supplementary material for: Perceived stigma and depression among the HIV-positive adult people in Ethiopia: A systematic review and meta-analysis
Source: PLoS One. 2024 Jun 3;19(6):e0302875. doi: 10.1371/journal.pone.0302875 (PMC11146698; doi:10.1371/journal.pone.0302875)
Supplement: Attachment — Submitted filename: Reviewer Comment.docx [file pone.0302875.s003.docx]

Review for **Plose one**

**Title**: Perceived stigma and depression among the HIV-positive adult people in Ethiopia: a
Systematic review and meta-analysis

**Manuscript number**: PONE-D-22-30518

**Thanks, dear editor for your invitation to review this work.**

1. The abstract section needs intensive revision and the recommendations should be based on the findings. The abstract seems grammatically problem and I think the software STATA 16 version is not done currently how to use this software and Please rewrite the abstract section based on Plose journal format
2. **Introduction:**
3. **Result**

- Just focus on Pooled prevalence not sample size only

Neither the figure nor the table were watched.

4. **Conclusion**

The conclusion should clearly show the major findings and the authors are expected to recommend based on modifieable variables.
